# Do Perceptions of Empowerment Affect Glycemic Control and Self-Care Among Adults with Type 2 Diabetes?

**DOI:** 10.5539/gjhs.v7n5p80

**Published:** 2015-02-24

**Authors:** Melba Sheila D’Souza, Subrahmanya Nairy Karkada, Nancy P. Hanrahan, Ramesh Venkatesaperumal, Anandhi Amirtharaj

**Affiliations:** 1 Adult Health and Critical Care, College of Nursing, Sultan Qaboos University, Muscat, Sultanate of Oman; 2 Department of Business Studies, Higher College of Technology, Al Khuwair, Oman; 3 University of Pennsylvania School of Nursing, Center for Health Outcomes and Policy Research Philadelphia, USA

**Keywords:** diabetes empowerment, type 2 diabetes mellitus, nursing, self-efficacy, self-care management, glycosylated hemoglobin, patient education

## Abstract

**Background:**

The Arab adult with T2DM is understudied with less known facts about the perception of empowerment and its relationship with self-care and glycemic control.

**Purpose:**

The purpose of this study was to determine the extent to which perception of empowerment by Arab adults living with Type 2 Diabetes Mellitus (T2DM) was associated with better glycemic control and self-care management.

**Methods:**

A cross-sectional descriptive study was led among 300 Arab adults living in Oman with T2DM in an outpatient diabetes clinic. The Diabetes Empowerment Scale (DES), glycosylated haemaglobin (HbA1c) and Body mass index was assessed. The DES was found to be valid and reliable for the population. ANOVA, Regression analysis, and Structural equation modeling was used for analysis.

**Results:**

The composite score and three subscales of DES were a significant and strong predictor of good glycemic control among Omani adults with T2DM (p<0.001). Age, education, duration of DM, prior DM education program and medications were significantly associated with DES.

**Conclusion:**

Diabetes nurse educators engaged in the care of adults with T2DM should assess self-empowerment and tailor interventions to increase empowerment for better glycemic control. Patient empowerment plays an essential role in maintaining self-care behaviours and HbA1c.

## 1. Introduction

Diabetes mellitus (DM) is a public health problem affecting millions of individuals, families, and communities worldwide. The World Health Organization predicts that diabetes mellitus (DM) will be the 7^th^ leading cause of death in 2030 (Alwan, 2011). Type 2 diabetes mellitus (T2DM) comprises 90-95% of all diabetes diagnoses among adults (Cox & Edelman, 2009) and is associated with high risk of complications, premature death, reduced quality of life (Williams, Walker, Smalls, Campbell, & Egede, 2014) and significant health care costs (Fowler 2008). T2DM incidence is predicted to grow along with the medical and economic burden of the disease indicating an urgent need for prevention of complications and novel interventions.

Since 1991, the prevalence of T2DM increased 15.4% among Arab Omani adults residing in Oman and over 20 years of age (Al-Lawati, Al Riyami, Mohammed, & Jousilahti, 2002; Ministry of Health, 2008). Improved living standards and socioeconomic conditions are thought to be associated with increased consumption of refined sugar, dried and evaporated whole milk, fast food, refined sugar, saturated fat, chicken, cheese, and chocolate products (Al-Lawati, Mabry, & Mohammed, 2008). Similar to other countries around the world, T2DM is growing at epidemic proportions among Omani adults (Aanstoot, 2009; Al-Lawati, Barakat, Al-Lawati, & Mohammed, 2008) with corresponding increases in complications associated with T2DM such as depression, loss of sight, limb amputations, infections, and early death (Williams, Walker, Smalls, Campbell, & Egede, 2014).

Although research is limited for Arab adults with T2DM, abundant research shows that educating individuals about diabetes treatment and self-care management—including drug therapy, appropriate risk factor control, and screening for diabetes-related complications—are cost-effective interventions that reduce the burden of diabetes and improve the quality of care on a large-scale basis. Empowerment perceptions are driven by culture and social norms. Research shows that patients who perceive they are empowered to self-manage their diabetes are more likely to be adherent with treatment and have better outcomes. The purpose of this study is to describe the Arab adult with T2DM and to understand the extent to which perceived empowerment and self-efficacy are related to better glycemic control.

**Figure 1:**
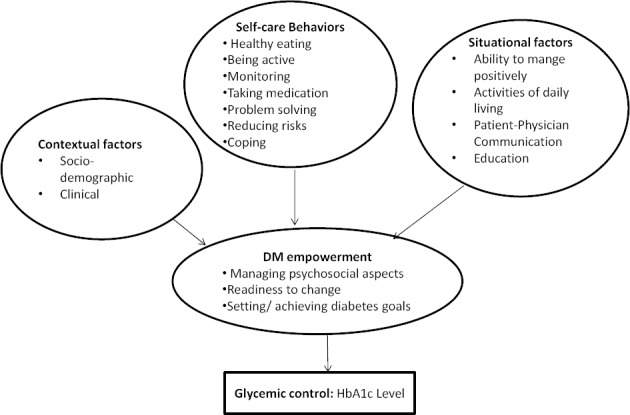
Diabetes empowerment model among Omani adults with T2DM

The Diabetes Empowerment Conceptual (DEC) framework (Figure 1) suggests that perception of empowerment may underlie effective diabetes self-management and thus better glycemic control (Figure 1). The DEC includes three constructs 1) Managing the Psychosocial Aspects of Diabetes; 2) Assessing Dissatisfaction and Readiness to Change; and 3) Setting and Achieving Diabetes Goals. Individuals with T2DM are empowered to prepare for change, set appropriate goals and handle day-to-day psychosocial stressors. Individuals, for example, who perceived empowerment might manage calories and exercise because they felt empowered with the knowledge to choose to control glucose levels thereby improving their health. Studies have shown that a greater sense of empowerment and self-efficacy is an antecedent to motivation to self-care.

### 1.1 Aim

Do perceptions of empowerment affect glycemic control and self-care management among adults living with Type 2 Diabetes Mellitus in Oman?

## 2. Method

### 2.1 Design

A cross-sectional descriptive design and structural equation modeling was used to determine relationships between perceived empowerment among Omani adults with T2DM and glycemic control.

### 2.2 Sampling Procedures

Starting June 2010, participants were selected from a clinic roster of patients with T2DM at an outpatient clinic that was located within a public hospital in Oman. Participants were included in the study if they were age 20 years or older, had a physician-determined diagnosis of type 2 diabetes, intact cognition, perceptual, sensory and communication ability.

### 2.3 Sample Size

For structural equation modeling (SEM), sample size was determined by power analysis based on root mean square error of approximation (RMSEA) (MacCallum, Browne, & Sugawara, 1996). The RMSEA was set at 0.05 and 0.08 for null and alternative models and 300 samples were found to be adequate for SEM (Steiger, 1990). A sample size of 330 was considered acceptable for this study to account for attrition.

### 2.4 Ethics

The study was approved by the Research and Ethics Committee at the Sultanate Qaboos University, College of Nursing. Participants were provided a written explanation of the purpose of the study and benefits and potential risks of participating. They were guaranteed confidentiality and were assured of voluntary withdrawal from the study at any time without any adverse consequences. Once consented, participants met with a diabetes nurse educator who administered the study survey. The completed study questionnaires were sealed in a closed envelope. Other data (lab value) was collected by the Diabetes Nurse Educator from the patient’s record. Of the 350 who met study criteria, 300 gave informed consent and provided complete data that were used in the analyses.

### 2.5 Measurements

***Demographic Characteristics*** were collected by the Diabetic Nurse: age, gender, formal education, smoking, duration of T2DM diagnosis, and the presence of a formal diabetes education.

***Diabetes Empowerment Scale (DES).*** The DES was administered twice with a 2-week interval to evaluate item reliability, stability, clarity and readability. The DES included 28 items that measure the psychosocial self-efficacy of people with diabetes and contains three subscales: Managing the Psychosocial Aspects of Diabetes subscale (α= 0.93) with 9 items; Assessing Dissatisfaction and Readiness to Change subscale (α = 0.81) with 9 items; and Setting and Achieving Diabetes Goals subscale (α=0.91) with 10 items. Participants responded to six items on a 5-point Likert-type scale ranging from 1 (*strongly disagree*) to 5 (*strongly agree*). Higher scores indicated that participants more frequently used empowerment actions and perceived higher levels of empowerment (Robert M Anderson, Funnell, Fitzgerald, & Marrero, 2000). The tool was found to be reliable.

The Content Validity Index (CVI) of the scale was calculated by dividing the number of items rated 3 or 4 by the total number of items. The CVI for DES was 0.90, which indicated that it was acceptable for use.

***Body Mass Index (BMI)*** was calculated using the World Health Organization calculation based on self-report of height and weight and calculated as weight in kilograms divided by the square of height in metres. BMI (kg/m^2^) = weight (kg)/[height (m^2^)]. and defined categories of BMI (World Health Organization, 2006). Overweight and obesity were defined as: *underweight*: BMI<18.5 kg/m^2^, *normal weight*: BMI 18.5–24.9 kg/m^2^, *overweight* (pre-obese): BMI 25–29.9 kg/m^2^ and *Obese*: BMI>30 kg/

***Hemaglobin A1C.*** HbA1Cvalues were categorized into 1) good glycemic control if HbA1C values are <7% and 2) poor glycemic control, if HbA1C values are >7% (American Diabetes Association, 2007).

## 3. Results

### 3.1 Recruitment

There was a 90.9% response rate among 330 eligible participants and 300 participants agreed to participate in the study.

### 3.2 Data Analysis

Univariate and bivariate statistics showed demographic characteristics, calculated mean, median, and range of the items of the DEC, BMI, and HbA1C using the Statistical Program for the Social Sciences. A confidence value of 95% and probability of p <0.05 was considered significant.

#### 3.2.1 Demographic and Clinical Characteristics (Table 1)

**Table 1:** Sample characteristics, glycemic control and significance among adults with T2DM, N=300

Characteristics	Categories	Good control n	%	Poor control n	%	Total N	%	DES p value
Age (years)	30-39	24	51.1	23	48.9	47	16	0.000*
	40-49	52	50.5	51	49.5	103	34	
	50-59	36	39.1	56	60.9	92	31	
	60 & above	26	44.8	32	55.2	58	19	
Gender	Male	54	37.8	89	62.2	143	48	0.396
	Female	84	53.5	73	46.5	157	52	
Education	Until 8^th^ grade	56	47.9	61	52.1	117	39	0.000*
	High school	51	54.3	43	45.7	94	31	
	Diploma/Technical	31	10.3	58	67.4	89	30	
Prevents activities of daily living	Never	43	39.8	65	60.2	108	36	0.000*
	Moderately	74	47.4	82	52.6	156	52	
	Mostly	21	58.3	15	41.7	36	12	
Ability to manage positively	Moderate ability	95	31.7	97	32.3	192	64	0.000*
	Good ability	43	14.3	65	21.7	108	36	
Duration of diabetes (years)	0-9	57	50.9	55	49.1	112	37	0.000*
	10-19	68	47.2	76	52.8	144	48	
	20 & above	13	29.5	31	70.5	44	15	
Diabetes education program	No	54	47.0	61	53.0	115	38	0.000*
	Yes	84	45.4	101	54.6	185	62	
Medications	Oral Hypoglycemics	109	48.7	107	51.3	216	75	0.000*
	Oral Hypoglycemics and insulin	29	9.7	55	18.3	84	25	
Body mass index	< 18.5 - Underweight	3	37.5	5	62.5	8	3	0.118
	18.5 - 24.9 - Healthy weight	87	43.1	115	56.9	202	67	
	25 - 29.9 - Overweight	48	53.3	42	46.7	90	30	

Note.

* p<0.001 level of significance using ANOVA. HbA1C (glycosylated haemaglobin) < 7% is good glycemic control, HbA1C > 7% is poor glycemic control. DM: Diabetes Mellitus, DES: Diabetes empowerment scale.

One-third of the adults with T2DM were aged 40-49 years (34%), of which half of the percentage had uncontrolled HbA1C>7% (49.5%); 46.5% of the females had uncontrolled HbA1C(>7%) compared to the men (62.2%); 45% of the adults with T2DM were tobacco users, of which 60.3% had uncontrolled HbA1C (Table 1). Nearly one-third of the adults had education until 8^th^ grade (39%), high school (31%) and diploma (30%). Nearly half of the adults (48%) lived with T2DM for 10-19 years, of which 52.8% had uncontrolled HbA1c (Table 1). Nearly 52% expressed that diabetes prevented their activities of daily living, and 64% reported that they had positive attitude and ability to manage diabetes. More than half of the adults (62%) were exposed to diabetes education program, of which 45.4% had controlled HbA1C. Most of the adults (75%) were on oral hypoglycemic agents (OHA), of which 48.7% had controlled HbA1C. More adults (67%) with T2DM showed healthy body mass index (BMI), of which 43.1% showed controlled HbA1C. 53.3% of the adults who were overweight (30%) showed controlled HbA1C.

Age, education, duration of DM, prior DM education program, medications was significantly associated with DES (Table 1). The perception of DM prevents activities of daily living and ability to manage DM positively was also significantly associated with DES.

#### 3.2.2 Global Diabetes Empowerment and Regression Analysis (Table 2)

**Table 2:** Diabetes empowerment scale (DES) among T2DM and regression analysis, N = 300

	Percentage of agreement with sub-dimensions of DES									Regression analysis		

Diabetes empowerment scale (DES)	Strongly agree		Agree		Disagree		Strongly disagree		Mean	B Coefficient	Std. Error.	p value
Sub-dimensions	n	%	n	%	n	%	n	%				

Managing psychosocial aspects of diabetes	13	4.33	107	35.67	59	19.67	121	40.33	3.07	.630	.026	0.001*
Assessing dissatisfaction/readiness to change	3	1.00	33	11.00	228	76.00	36	12.00	3.00	.369	.015	0.001*
Setting/achieving diabetes goals	23	7.67	109	36.33	60	20.00	108	36.00	3.15	.614	.025	0.001*

Overall DES	4	1.33	82	27.33	149	49.67	65	21.67	3.07	.657	.027	0.001*

Note.

* p<0.001 level of significance using regression analysis.

Nearly 7.67% of the adults with T2DM strongly agreed to Setting and achieving goals, e.g. choosing realistic diabetes goals (Table 2). One-third of the adults with T2DM were able to Set and achieve goals (36.33%) and Manage psychosocial aspects (35.67%), e.g. positive ways of coping with diabetes-related stressed. Most of the adults agreed that they were dissatisfied and not ready to change (76%), e.g. dissatisfied with areas of taking care of diabetes. Some of the adults strongly disagreed with ability to manage psychosocial aspects (40.33%) and Setting goals (36%). The highest mean score among the 3 DES sub-dimensions was Setting and Achieving Diabetes Goals subscale (mean=3.15+0.99). Global DES and the three sub-dimensions of DES (p<0.001) were highly significant among adults with T2DM.

#### 3.2.3 Diabetes Empowerment Sub-Dimensions (Table 3)

**Table 3:** Diabetes empowerment sub-dimensions among adults with T2DM, N = 300

Diabetes empowerment process	Strongly agree		Agree		Disagree		Strongly disagree		Neutral	

	n	%	n	%	n	%	F	%	n	%
Managing the Psychosocial Aspects of Diabetes										

know the positive ways I cope with diabetes-related stress.	35	11.67	77	25.67	38	12.67	137	45.67	13	4.33
can cope well with diabetes-related stress.	33	11.00	101	33.67	39	13.00	119	39.67	8	2.67
know where I can get support for having and caring for my diabetes.	33	11.00	79	26.33	51	17.00	118	39.33	19	6.33
can ask for support for having and caring for my diabetes when I need it.	21	7.00	93	31.00	36	12.00	142	47.33	8	2.67
can support myself in dealing with my diabetes.	17	5.67	95	31.67	32	10.67	151	50.33	5	1.67
know what helps me stay motivated to care for my diabetes.	31	10.33	95	31.67	26	8.67	144	48.00	4	1.33
can motivate myself to care for my diabetes.	29	9.67	98	32.67	28	9.33	141	47.00	4	1.33
know enough about diabetes to make self-care choices that are right for me.	34	11.33	100	33.33	27	9.00	134	44.67	5	1.67
know enough about myself as a person to make diabetes care choices that are right for me.	21	7.00	105	35.00	31	10.33	135	45.00	8	2.67
Assessing Dissatisfaction and Readiness to Change										

know what part(s) of taking care of my diabetes that I am satisfied with.	15	5.00	65	21.67	38	12.67	141	47.00	41	13.67
know what part(s) of taking care of my diabetes that I am dissatisfied with.	17	5.67	164	54.67	29	9.67	66	22.00	24	8.00
know what part(s) of taking care of my diabetes that I am ready to change.	22	7.33	65	21.67	29	9.67	174	58.00	10	3.33
know what part(s) of taking care of my diabetes that I am not ready to change.	5	1.67	182	60.67	27	9.00	61	20.33	25	8.33
can tell how I’m feeling about having diabetes.	37	12.33	76	25.33	57	19.00	114	38.00	16	5.33
can tell how I’m feeling about caring for my diabetes	34	11.33	68	22.67	37	12.33	135	45.00	26	8.67
know the ways that having diabetes causes stress in my life.	33	11.00	69	23.00	36	12.00	144	48.00	18	6.00
know the negative ways I cope with diabetes-related stress.	11	3.67	121	40.33	43	14.33	89	29.67	36	12.00
how care am able to figure out if it is worth my while to change how I take care of my diabetes.	26	8.67	97	32.33	32	10.67	136	45.33	9	3.00
Setting and Achieving Diabetes Goals	n	%	n	%	n	%	F	%	n	%

can choose realistic diabetes goals.	38	12.67	93	31.00	33	11.00	130	43.33	6	2.00
know which of my diabetes goals are most important to me.	34	11.33	113	37.67	27	9.00	123	41.00	3	1.00
know the things about myself that either help or prevent me from reaching my diabetes goals.	35	11.67	107	35.67	28	9.33	127	42.33	3	1.00
can come up with good ideas to help me reach my goals.	33	11.00	114	38.00	31	10.33	119	39.67	3	1.00
am able to turn my diabetes goals into a workable plan.	27	9.00	116	38.67	29	9.67	122	40.67	6	2.00
can reach my diabetes goals once I make up my mind.	20	6.67	110	36.67	37	12.33	123	41.00	10	3.33
know which barriers make reaching my diabetes goals more difficult.	46	15.33	89	29.67	39	13.00	121	40.33	5	1.67
can think of different ways to overcome barriers to my diabetes goals	28	9.33	69	23.00	79	26.33	123	41.00	1	0.33
can try out different ways of overcoming barriers to my diabetes goals.	60	20.00	62	20.67	49	16.33	126	42.00	3	1.00
am able to decide which way of overcoming barriers to my diabetes goals works best for me.	34	11.33	74	24.67	48	16.00	136	45.33	8	2.67

One-third to quarter percentage of the adults agreed that they were able to Manage their psychosocial aspects of DM (25.67%-35%) compared to those who strongly disagreed (39.33%-50.33%) (Table 3). Many adults with T2DM agreed they were able to Assess dissatisfaction and readiness to change (21.67%-60.67%) compared those who disagreed (20.33%-58%) with them. Some of the adults agreed that they were able to Set and achieve diabetes goals (20.67%-38.67%) compared to those who strongly disagreed (39.67%-45.33%). Hence perceptions of empowerment affected glycemic control.

### 3.3 Structural Equation Modelling

#### 3.3.1 Testing of Hypotheses

H0_1:_ There is positive hypothetical relationship between Psychosocial factors, Readiness to change and Setting goals.

The results show that Chi-square = 17415.6, degrees of freedom = 6, and probability level = 0.0001 (Table 5)

**Table 4:** Regression weights and lisrel maximim likelihood estimates

Latent Variable		Measured Variables	Estimates	SE	R^2^	CR	P
OVERALL	<---	PSY	3.152	.057	.75	54.968	0.001
OVERALL	<---	RDN	3.004	.028	.67	108.049	0.001
OVERALL	<---	GLS	3.068	.061	.41	50.369	0.001

p<0.001, significant at 1% level.

**Table 5:** Model fit indices

Sl. No	Model Fit Indices	Calculated Value	Acceptable Threshold Levels
1	Comparative Fit Index(CFI)	0.562	0-1
2	Normed Fit Index (NFI)	0.726	0-1
3	Relative Fit Index (RFI)	0.628	0-1
4	Incremental Fit Index (IFI)	0.825	0-1
5	Parsimonious Normed Fit Index (PNFI))	0.682	0-1
6	Parsimony Comparative Fit Index (PCFI)	0.564	0-1
7	Tucker Lewis Index (TLI)	0.728	0-1
8	Root Mean Squared Error of Approximation (RMSEA)	0.03	0.05 or less would indicate a close fit of the model

#### 3.3.2 Regression Weights and Lisrel Maximim Likelihood Estimates (Table 4)

All the manifest variables (Psychosocial, Readiness to change, and Setting goals) are influenced with the latent variable (Overall DES) of successful operation and also have positive relationship with the significance at 1% and 5 %. Table 4 indicates that the regression coefficient of the exogenous variables. The critical ratio of all the manifest variables is above the table value of 2.962 and it is significant at 1%.

#### 3.3.3 Model Fit Indices (Table 5)

Table 5 conveys that the model fit indices of the variables. The entire test has the range of 0 to 1. The comparative fit index (CFI) scored 0.562, normed fit index (NFI) scored 0.726, relative fit index (RFI) scored 0.628, incremental fit index (IFI) scored 0.825, parsimonious normed fit Index (PNFI) scored 0.682, parsimony comparative fit index (PCFI) scored 0.564, Tucker Lewis index (TLI) scored 0.728, and the Root Mean Squared Error of Approximation (RMSEA) secured 0.03 that indicates a close fit of the model.

## 4. Discussion

Some adults with T2DM reported that they were able to manage their psychosocial aspects of DM related to making right diabetes care choices, coping with diabetes-related stress, and knew about diabetes to make self-care. Some adults perceived good ability to positively fit self-management in their daily life perceived lower HbA1c level. The dimension of the ‘setting and achieving diabetes goal’ was reported to be the most important empowerment domain (Tol et al., 2012). Adults who reported good health had high scores on the Swe-DES-23 scale (Leksell, et al., 2007) and Chinese version DES (Mei-Fang Chen et al., 2011). This study shows that empowerment is a crucial variable in the self-care management and glycemic control among adults with T2DM.

The conceptual framework was supported by the empowered adults who managed their diabetes and had better glycemic control than participants who had low scores on the DES. This means that participants who were empowered and actively managing their diabetes had better metabolic control. This study showed a significant relationship between the participants’ perceptions of Managing the Psychosocial Aspects of Diabetes, Readiness to change and Achieving goals and HbA1c. There a significant association between empowerment and positive metabolic control, self-efficacy and self-care behaviours (Peña-Purcell, Boggess, & Jimenez, 2011). This is similar to other studies related to self-care behaviours and psyschosocial factors that have influenced metabolic control compared to those with lower HbA1c (Cosansu & Erdogan, 2014; Mahjouri, Arzaghi, Qorbani, Nasli-Esfahani, & Larijani, 2011).

In contrast poor empowerment was due to inadequate management of psychosocial aspects related to knowledge of treatment and self-management, difficulty in readiness to change related to social (D’Souza et. al., 2013), self-care behaviours, and poor goal setting related to plan of action for achieving diabetes targets in the study. Increased empowerment was influenced by social support, exposure to education, self-efficacy in managing psychosocial aspects. Adults with T2DM felt empowered in their self-care ability (Sigurdardottir & Jonsdottir, 2008). Other studies showed that open communication (Funnell et al., 2009), mutual participation, sufficient knowledge and skills (Musacchio et al., 2011) and decisions related to goals is important in the diabetes empowerment process(Kettunen, Liimatainen, Villberg, & Perko, 2006; Skinner et al., 2006).

There was higher level of empowerment among adults in the middle age group (40-49 years), moderate duration of DM (10-19 years), prior DM education and use of oral medications. Adults with T2DM felt empowered in their self-care ability (Sigurdardottir & Jonsdottir, 2008). Education was associated with global DES among Turkish adults with T2DM (p < 0.01) indicating greater perception of empowerment among those with higher education (Tol et al., 2013). Empowerment is strongly influenced by religion, faith, cultural and spirituality (Redfield, 2011), and social, emotional and family support (Song et al., 2012). Patients who perceive higher empowerment have higher success with self-management and glycemic outcomes. The strength of the findings should spur diabetes nurse educators to assume that patients who perceive higher empowerment engage in the active involvement, thereby necessitating individualized tailored interventions to increase empowerment among Omani adults with T2DM.

Limitation included socio-cultural restrictions that may have hampered free responses in self-reports among Omani adults. A dyadic interaction between adults and the nurse educators limits an understanding of empowerment.

## 5. Conclusion

A significant percentage of the adults did not have a good sense of empowerment. Determinants of empowerment (ability to manage positively, education, patient-physician communication, activities of daily living) can improve the self-care beahviours for active participation in self-care management among adults with T2DM. This study showed that Omani adults with T2DM were not empowerment with their self-care management to make informed decisions or control their illness. They had moderate knowledge about their illness and problem solving ability to improve self-care management aspects. Only some adults perceive self-efficacy and readiness to change and ability to set and achieve goals, resulting in improved self-care. They have active participation to make informed decisions, have a sense of self-control and self-efficacy to improve HbA1c.

Adults with T2DM must have insight into their own needs, and they need to have knowledge about diabetes and its self-care. Empowerment strategies should address the determinants of empowerment for active participation in self-care activities. Achieving these tasks provide a sense of gain and mastery of glycemic control which enhances self-efficacy. Thus empowerment process leads to increase perceived self-efficacy and self-management among Omani adults with T2DM.

Empowering adults with T2DM is an intervention strategy that diabetes nurse educators should place in their diabetes resource toolkit including e-health and e-literacy. This mutual relationship can enable patient empowerment, a key component of self-care. Adults with T2DM who actively collaborate in the decision-making process are able to achieve glycemic control. Empowerment promotes better HbA1c and self-care through healthy self-care behaviors, life style modification, and social-cultural factors among Omani adults with T2MD. Empowered adults with T2DM are capable of making appropriate self-care decisions that requires managing diabetes.
